# MitoQ in sepsis-associated acute lung injury: redox pharmacology, mechanistic evidence, and translational perspectives

**DOI:** 10.3389/fphar.2026.1975929

**Published:** 2026-09-30

**Authors:** Jiajia Yu, Yuyu An, Yanni Mi, Guoquan Wang, Bin Wang, Jiping Liu

**Affiliations:** 1 Department of Pharmacology, Shaanxi University of Chinese Medicine, Xianyang, China; 2 Key Laboratory of Pharmacodynamic Mechanism and Material Basis of Traditional Chinese Medicine, Shaanxi Administration of Traditional Chinese Medicine, Xianyang, China

**Keywords:** alveolar-capillary barrier, cGAS-STING, mitochondrial DNA, mitochondrial oxidative stress, MitoQ, NLRP3 inflammasome, pyroptosis, sepsis-associated acute lung injury

## Abstract

Sepsis-associated acute lung injury (SALI) is characterised by mitochondrial oxidative stress, bioenergetic dysfunction, dysregulated inflammation, and disruption of the alveolar–capillary barrier. MitoQ is a mitochondria-targeted redox modulator with experimental evidence of mitochondrial and pulmonary protection, making it a candidate strategy for limiting oxidative injury in SALI. This critical narrative review evaluates the redox pharmacology of MitoQ, direct pulmonary and sepsis-related evidence, mechanistic links between mitochondrial damage and innate immune signalling, and the major requirements for clinical translation. Available studies show that MitoQ reduces mitochondrial reactive oxygen species and lipid peroxidation, preserves membrane potential and ATP production, and attenuates epithelial, endothelial, and alveolar–capillary barrier injury. Mechanistic studies further support links among mitochondrial dysfunction, abnormal mitochondrial DNA exposure, cGAS–STING signalling, NLRP3–GSDMD activation, pyroptosis, and pulmonary barrier failure. However, these downstream pathways have not yet been causally linked to MitoQ treatment in infection-associated SALI, and pulmonary or intramitochondrial drug exposure remains poorly defined. Human studies demonstrate oral feasibility and systemic redox pharmacodynamic activity but have not established pulmonary target engagement or clinical efficacy. Current evidence therefore supports MitoQ primarily as an upstream mitochondrial redox modulator with potential to preserve alveolar–capillary function. Future studies should prioritise post-onset treatment, pulmonary and mitochondrial target engagement, pathway-specific causal validation, and assessment of host defence to determine its translational potential in SALI.

## Graphical Abstract

**Figure:**
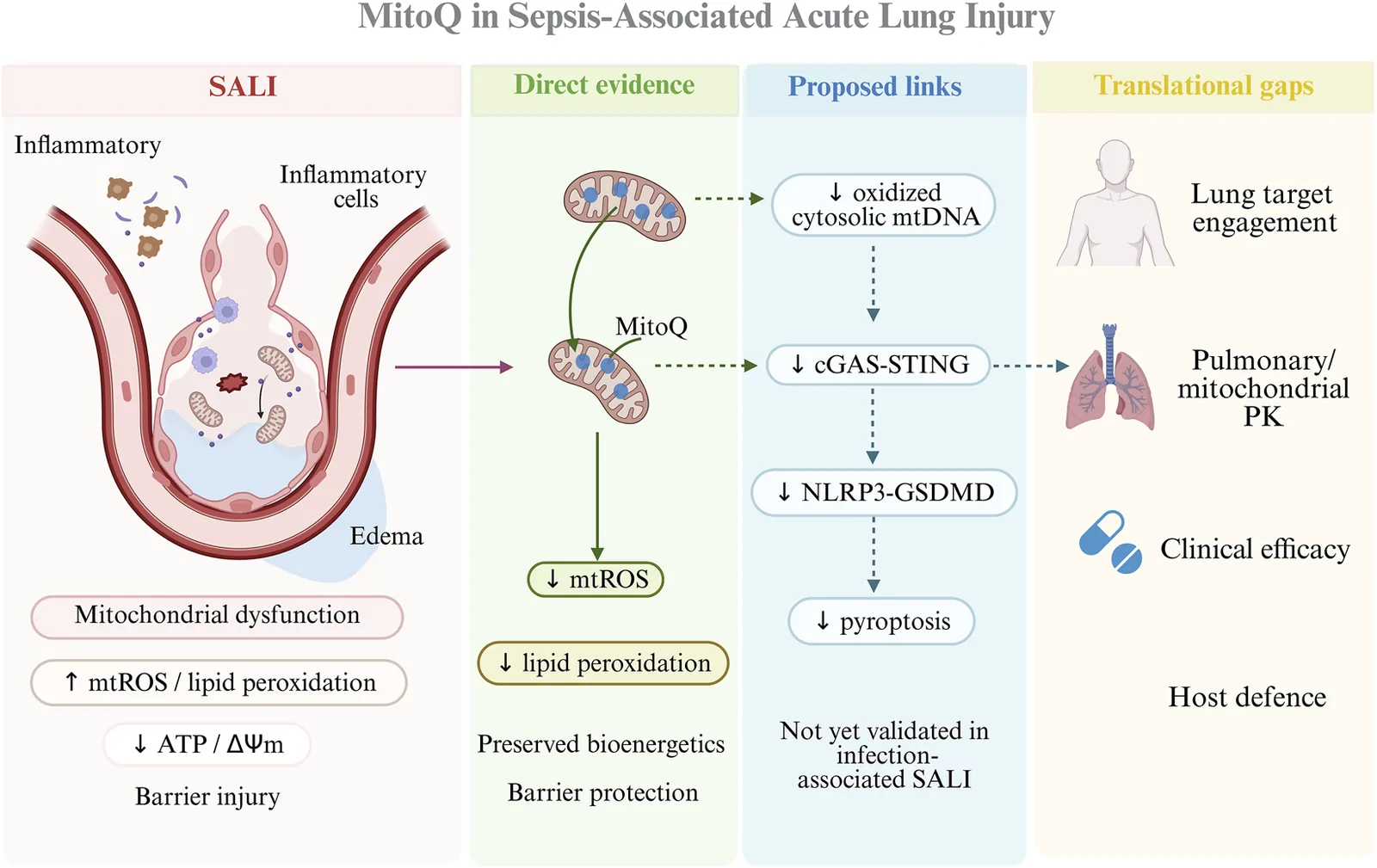

## 1 Introduction

Sepsis-associated lung injury encompasses pulmonary injury caused by sepsis and may meet contemporary diagnostic criteria for acute respiratory distress syndrome (ARDS), including the Berlin and New Global Definitions (Matthay et al., 2024; Ranieri et al., 2012). In this review, SALI is used as the broader term, whereas sepsis-associated ARDS (SA-ARDS) refers specifically to patients who fulfil established ARDS criteria. Disruption of the alveolar epithelium and pulmonary microvascular endothelium increases alveolar–capillary permeability, resulting in protein-rich pulmonary oedema, impaired gas exchange, and hypoxaemic respiratory failure. Management remains largely supportive, and no established pharmacological therapy directly restores alveolar–capillary barrier integrity. Mechanisms linking cellular injury to barrier failure therefore remain important therapeutic targets.

Mitochondrial dysfunction is a prominent feature of SALI. Inflammatory mediators, microbial products, tissue hypoperfusion, and metabolic stress impair respiratory-chain activity and oxidative phosphorylation. These changes promote mitochondrial membrane depolarisation, ATP depletion, mitochondrial reactive oxygen species (mtROS) accumulation, and lipid peroxidation. The resulting bioenergetic and oxidative stress can impair epithelial and endothelial function, disrupt junctional integrity, and limit cellular repair. Mitochondrial injury may also increase mitochondrial DNA (mtDNA) oxidation and abnormal mtDNA exposure through defective mitochondrial quality control. Circulating mtDNA has been associated with acute lung injury, ARDS, and adverse outcomes in patients with sepsis and severe trauma (Faust et al., 2020; Mao et al., 2021). Interpretation nevertheless depends on the compartment, oxidation state, and analytical form of mtDNA measured. Mitochondrial danger signals can activate innate immune and regulated cell-death pathways. Oxidised mtDNA can promote NLRP3 inflammasome activation (Shimada et al., 2012), while cytosolic DNA sensing through cGAS–STING has been linked to NLRP3 activation, caspase-1 maturation, gasdermin D (GSDMD) cleavage, and pulmonary injury in lipopolysaccharide-based models (Ning et al., 2020). GSDMD cleavage generates a pore-forming N-terminal fragment that promotes membrane permeabilisation, inflammatory mediator release, and lytic cell death (Shi et al., 2015). These pathways should not be viewed as an obligatory linear cascade. cGAS–STING, inflammasomes, and gasdermins can be activated by mitochondrial and non-mitochondrial signals. Alveolar–capillary barrier failure can also arise through bioenergetic failure, apoptosis, ferroptosis, junctional remodelling, and cytoskeletal disruption. mtDNA is therefore more appropriately regarded as an amplifier of mitochondrial danger signalling than as the sole driver of SALI.

MitoQ is a mitochondria-targeted redox modulator composed of a ubiquinone moiety linked to a lipophilic triphenylphosphonium cation. Its membrane potential-dependent accumulation near the inner mitochondrial membrane enables local redox modulation and may preserve mitochondrial structure and bioenergetics. In experimental acute and sepsis-associated lung injury, MitoQ reduces mtROS accumulation, preserves mitochondrial membrane potential, ATP production, and ultrastructure, and attenuates pulmonary oedema, junctional protein loss, epithelial and endothelial injury, and microvascular hyperpermeability (Cen et al., 2021; Hou et al., 2021; Li et al., 2019; Sun et al., 2025). These findings support mitochondrial and alveolar–capillary barrier protection. However, they do not establish that these effects are mediated by reduced mtDNA exposure or suppression of cGAS–STING–NLRP3–GSDMD signalling. Human evidence remains limited. A small randomised trial in septic shock demonstrated systemic redox pharmacodynamic activity without evidence of clinical efficacy (Moradbaki et al., 2026).

MitoQ is not the only mitochondria-directed intervention investigated in experimental lung injury. MitoTEMPO, another TPP^+^-based compound, couples mitochondrial targeting to a nitroxide redox moiety and has attenuated mitochondrial oxidative stress and lung injury in experimental ALI (Wang X. et al., 2024; Yao et al., 2026). Elamipretide (SS-31), in contrast, is a mitochondria-targeting tetrapeptide that interacts with cardiolipin and supports inner-mitochondrial-membrane structure and bioenergetics; protective effects have likewise been reported in LPS-induced lung injury (Elmorsy et al., 2026; Luo et al., 2024). These strategies therefore address overlapping features of mitochondrial dysfunction through pharmacologically distinct mechanisms. Experimental ALI studies indeed support pulmonary effects of both MitoTEMPO and SS-31/elamipretide.

Against this broader therapeutic landscape, MitoQ is particularly suitable for a drug-specific critical appraisal not because it has been established as superior to other mitochondria-targeted interventions, but because its evidence base spans mitochondrial-targeting pharmacology, direct pulmonary epithelial and endothelial studies, polymicrobial sepsis models, and human septic-shock pharmacodynamic data. This breadth permits evaluation of a single compound across mitochondrial target engagement, alveolar–capillary barrier protection, exposure-related constraints, mechanistic uncertainty, and clinical translation. Recent reviews have examined mitochondrial dysfunction in ARDS from broader perspectives, including immunometabolic reprogramming, metabolic resilience, molecular phenotypes, and mitochondria-directed therapies (Srinivas et al., 2026). However, a focused appraisal of the drug-specific evidence for MitoQ in infection-associated lung injury is lacking. This review evaluates the evidence for pulmonary target engagement, an mtDNA-linked causal mechanism, and further development of MitoQ for infection-associated SALI. Direct pulmonary and sepsis-related MitoQ studies are distinguished from extrapulmonary mechanistic evidence, pulmonary studies of other mitochondrial interventions, and human feasibility data. This evidence-tiered approach distinguishes directly supported effects from inferential mechanisms and identifies the evidence required for causal validation and clinical translation (Figure 1).

**FIGURE 1:**
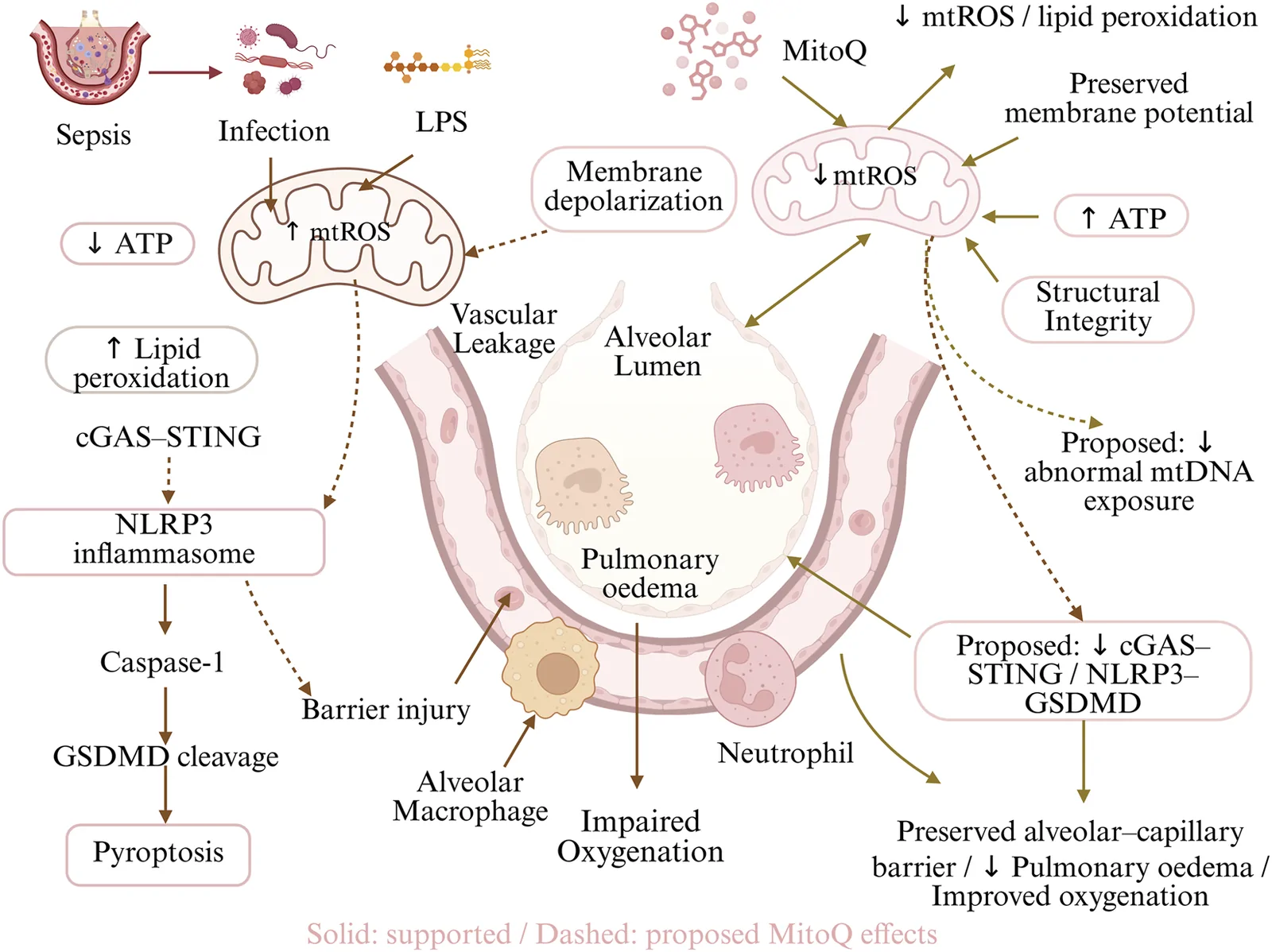
Evidence-based framework for the proposed actions of MitoQ in SALI. Sepsis-associated inflammatory stress promotes mitochondrial oxidative injury, bioenergetic failure, and disruption of the alveolar–capillary barrier. Pulmonary studies directly support reductions in mtROS and lipid peroxidation, preservation of mitochondrial membrane potential and ATP production, and attenuation of barrier injury by MitoQ. Reduced abnormal mtDNA exposure and downstream modulation of cGAS–STING and NLRP3–GSDMD signalling remain indirectly supported or unvalidated in infection-associated SALI. Solid arrows indicate directly supported effects, whereas dashed arrows indicate proposed mechanistic links.

## 2 Literature search strategy and evidence selection

This study was conducted as a structured critical narrative review rather than a systematic review or meta-analysis. PubMed/MEDLINE and the Web of Science Core Collection were searched for English-language publications from database inception to September 2026. Search terms included “MitoQ” or “mitoquinone” together with terms related to sepsis, acute lung injury, acute respiratory distress syndrome, mitochondrial oxidative stress, mitochondrial DNA, cGAS–STING, NLRP3, GSDMD, pyroptosis, mitochondrial quality control, and pulmonary barrier dysfunction. The searches identified 6,718 records, of which 5,407 remained after de-duplication. Titles and abstracts were screened for relevance, and 144 articles were retained for detailed assessment. Of these, 60 were excluded because they involved non-pulmonary or non-sepsis settings without sufficient mechanistic relevance, duplicated stronger or more directly relevant evidence, or provided only peripheral contextual information. Eighty-four database-derived publications were retained.

Studies were prioritised if they directly evaluated MitoQ in pulmonary, acute lung injury, or sepsis-related models. Extrapulmonary MitoQ studies were included when they provided relevant evidence on mitochondrial oxidative injury, mtDNA signalling, mitochondrial quality control, regulated cell death, barrier dysfunction, or human safety and translational relevance. Pulmonary or sepsis-related studies not directly testing MitoQ were included when they provided mechanistic bridging evidence relevant to the proposed pathways. Studies not relevant to MitoQ pharmacology, SALI-associated pulmonary injury, or the predefined mechanistic framework were excluded.

The final review included 105 unique references, of which 84 were identified from the database search pool and 21 through targeted citation tracking and supplementary searches. Evidence was qualitatively classified as direct pulmonary or sepsis-related MitoQ evidence, extrapulmonary MitoQ evidence, mechanistic bridging evidence, or human feasibility/pharmacodynamic evidence.

## 3 Redox pharmacology and exposure-related limitations of MitoQ

### 3.1 Mitochondrial targeting and redox activity

MitoQ consists of a ubiquinone moiety linked to a lipophilic triphenylphosphonium cation through a 10-carbon alkyl chain. The delocalized positive charge facilitates passage across phospholipid membranes and promotes membrane potential-dependent accumulation within mitochondria, whereas the ubiquinone moiety provides redox activity (Smith et al., 2012; Smith and Murphy, 2010). Because the mitochondrial matrix is generally 150–180 mV more negative than the cytosol, MitoQ can achieve substantially higher concentrations within mitochondria than in cytosolic or extracellular compartments (Feniouk and Skulachev, 2017; Fields et al., 2023). Within mitochondria, MitoQ partitions predominantly at the lipid–water interface of the inner mitochondrial membrane, close to sites of respiratory-chain oxidant production and lipid radical formation. Mitoquinone is reduced by the respiratory chain to the ubiquinol form, which reacts with lipid peroxyl radicals and interrupts lipid peroxidation. Subsequent reoxidation allows MitoQ to re-enter the local redox cycle (Farshad and Wehrend, 2025; Feniouk and Skulachev, 2017; Fields et al., 2023). This activity may protect inner-membrane lipids, preserve membrane organisation, and support oxidative phosphorylation. These effects are potentially relevant because sustained respiratory-chain dysfunction damages phospholipids, iron–sulphur clusters, respiratory-chain proteins, and mtDNA, thereby reinforcing oxidative stress and bioenergetic failure (Murphy, 2016). Foundational studies demonstrated rapid, membrane potential-dependent mitochondrial accumulation of MitoQ and protection against lipid peroxidation and oxidant-induced mitochondrial injury (Kelso et al., 2001). However, its effects vary according to the cellular context, injurious stimulus, local concentration, and extent to which mitochondrial oxidative damage contributes to disease pathogenesis. MitoQ is therefore more appropriately regarded as a mitochondria-targeted redox modulator than as a nonspecific cellular ROS scavenger or a broad inhibitor of regulated cell death.

### 3.2 Exposure, membrane potential, and dose-dependent constraints

The uptake and retention of MitoQ depend on cellular permeability, molecular lipophilicity, and the magnitude of the mitochondrial membrane potential. The triphenylphosphonium moiety drives electrophoretic accumulation across the inner mitochondrial membrane, whereas linker structure and hydrophobicity influence uptake kinetics and intramitochondrial distribution (Kelso et al., 2001; Ross et al., 2008). Consequently, the pharmacological behaviour of MitoQ in healthy, well-polarised mitochondria may not directly predict its behaviour in severely injured tissue. This issue is particularly relevant to SALI. Systemic hypoperfusion may reduce delivery to the injured lung, while pronounced mitochondrial depolarisation may limit organelle-specific accumulation. Pulmonary distribution, intracellular concentrations, and intramitochondrial exposure have not been quantified in infection-associated lung injury. Therapeutic activity may therefore depend not only on the administered dose but also on disease severity, organ perfusion, treatment timing, and the residual membrane potential of target-cell mitochondria. The effects of MitoQ are also concentration- and structure-dependent. Concentrations used in some *in vitro* studies may exceed those achievable *in vivo*. At higher concentrations, MitoQ and related alkyltriphenylphosphonium compounds lacking the ubiquinone group can suppress oxidative phosphorylation and induce compensatory glycolysis, indicating that the targeting cation, linker, and hydrophobic properties may alter mitochondrial metabolism independently of ubiquinone-mediated redox activity (Reily et al., 2013). Under selected respiratory conditions, reduced MitoQ may also undergo redox cycling and increase superoxide or hydrogen peroxide production (James et al., 2005). Although these observations should not be extrapolated directly to clinically relevant exposure, they demonstrate that MitoQ cannot be assumed to exert antioxidant effects under all experimental conditions. Accordingly, interpretation of experimental findings requires simultaneous consideration of administered dose, local drug concentration, mitochondrial membrane potential, submitochondrial distribution, respiratory substrate availability, and the cellular redox environment. Studies relying only on nominal extracellular concentrations or administered doses cannot determine whether a protective effect reflects adequate mitochondrial target engagement, whereas a neutral result cannot distinguish biological inefficacy from inadequate pulmonary delivery or impaired mitochondrial uptake.

### 3.3 Implications for infection-associated lung injury

By limiting mitochondrial oxidative injury, MitoQ may preserve mitochondrial function and reduce the generation of mitochondrial danger signals. Whether this translates into reduced mtDNA-dependent innate immune activation in infection-associated SALI remains unresolved. Because physiological mtROS contributes to antimicrobial signalling, excessive redox suppression may also compromise host defence (Figure 2).

**FIGURE 2:**
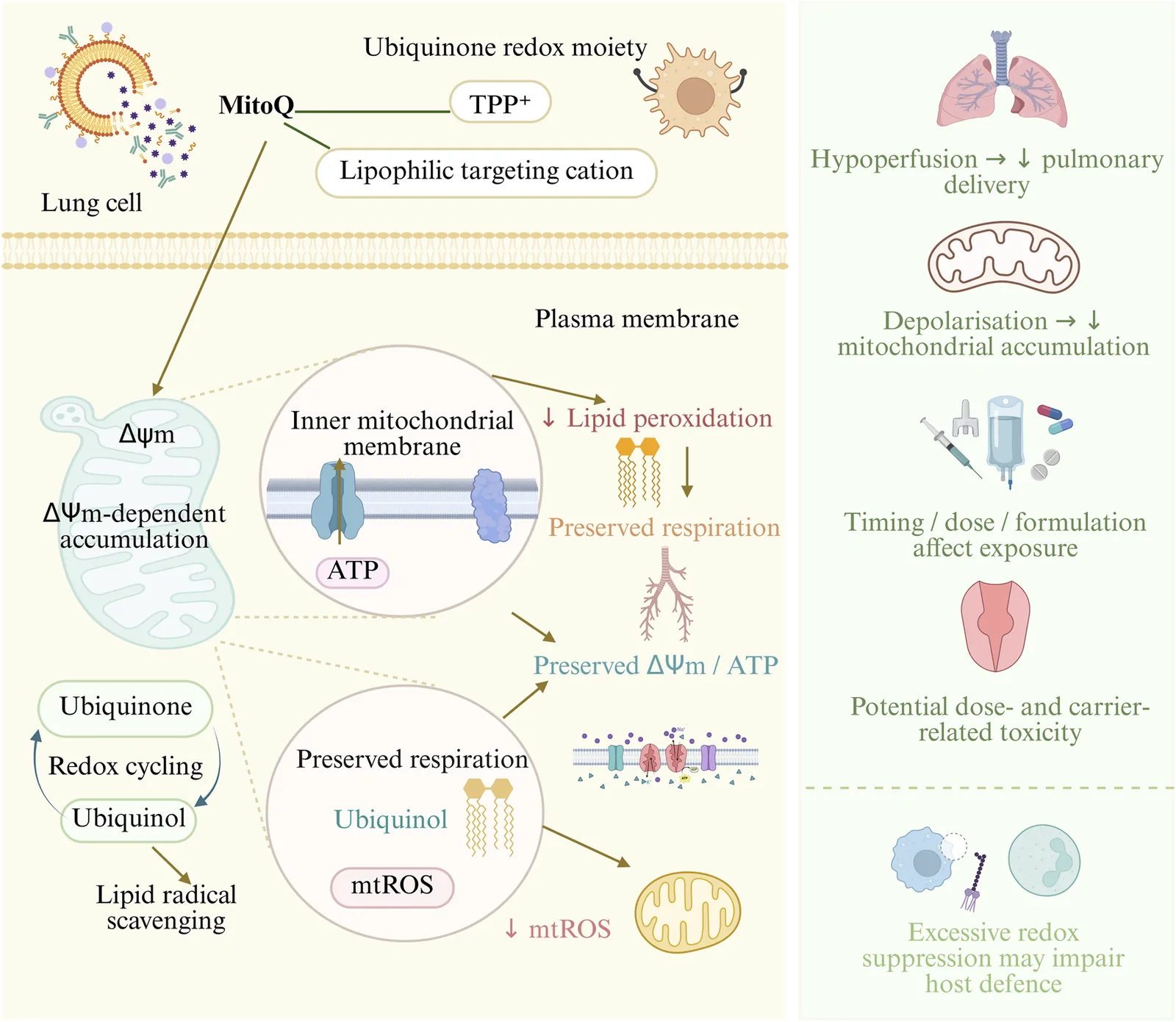
Mitochondrial targeting, redox activity, and potential translational constraints of MitoQ in SALI. The lipophilic TPP^+^ cation promotes membrane potential-dependent mitochondrial accumulation, while the ubiquinone moiety undergoes redox cycling near the inner mitochondrial membrane and limits lipid-peroxidation chain reactions. In SALI, pulmonary delivery and intramitochondrial accumulation may be constrained by hypoperfusion, mitochondrial depolarisation, treatment timing, and dose-dependent effects. Potential carrier-related effects and excessive suppression of physiological redox signalling also require evaluation. Dashed arrows indicate proposed or context-dependent effects that remain incompletely established in infection-associated SALI.

## 4 Direct evidence for MitoQ in acute and sepsis-associated lung injury

### 4.1 Direct pulmonary evidence

The alveolar–capillary barrier depends on the integrity of alveolar epithelial and pulmonary microvascular endothelial cells. In LPS-treated pulmonary microvascular endothelial cells, MitoQ reduced mtROS, preserved mitochondrial membrane potential and ATP production, and attenuated apoptosis and hyperpermeability; similar effects were observed *in vivo*, including reduced pulmonary vascular leakage. Disruption of Nrf2 or MafF weakened these effects, implicating Nrf2–MafF/antioxidant response element signalling (Cen et al., 2021). However, MitoQ was administered predominantly before LPS exposure, limiting therapeutic interpretation. The study also did not establish an mtDNA-dependent or pyroptotic mechanism, and the barrier protection may instead reflect redox preservation, reduced apoptosis, and junctional stabilisation. In an LPS-induced epithelial injury model, MitoQ activated Nrf2 signalling, limited Drp1-dependent mitochondrial hyperfission, reduced mtROS and membrane depolarisation, and suppressed cytochrome c release and caspase-3 activation (Hou et al., 2021). It also restored ZO-1 and occludin expression, whereas Nrf2 inhibition or Drp1 overexpression weakened these effects. Post-LPS administration reduced pulmonary oedema, inflammation, and epithelial injury *in vivo*, providing greater therapeutic relevance than prophylactic designs. However, the predominant use of A549 cells limits extrapolation to primary alveolar type II epithelium. In paraquat-injured A549 cells, MitoQ also attenuated epithelial injury and promoted MFN1/MFN2-mediated mitochondrial fusion (Liu et al., 2022). Although this model is not specific to SALI, it supports a role for mitochondrial dynamics in MitoQ-associated protection without establishing involvement of the mtDNA–cGAS–STING–NLRP3–GSDMD pathway.

### 4.2 Evidence from infection-associated and other lung-injury models

Direct evidence for MitoQ in polymicrobial sepsis is derived mainly from cecal ligation and puncture (CLP) models. In rats, post-CLP administration of MitoQ improved 24-h survival and reduced pulmonary oedema, inflammatory-cell infiltration, apoptosis, myeloperoxidase activity, and HMGB1 and IL-6 concentrations (Li et al., 2019). MitoQ also increased Akt, GSK-3β, and mTOR phosphorylation, while PI3K inhibition weakened these effects, implicating PI3K–Akt–GSK-3β–mTOR signalling in early pulmonary protection. The post-onset design increases therapeutic relevance, although the short follow-up precluded assessment of sustained survival and organ recovery. In a second CLP study, MitoQ reduced lung injury and bronchoalveolar lavage fluid protein leakage while improving oxygenation, respiratory-chain activity, ATP production, mitochondrial ultrastructure, and mtDNA copy number (Sun et al., 2025). Citrate synthase silencing attenuated several effects, supporting a contribution of mitochondrial metabolic homeostasis. However, treatment began 2 weeks before CLP, substantially limiting therapeutic interpretation. Preservation of mtDNA copy number also reflects mitochondrial genome abundance or stability rather than reduced mtDNA oxidation, cytosolic or extracellular release, or innate immune recognition. Less disease-specific inflammatory models provide additional evidence. In chronic ethanol-plus-LPS exposure, MitoQ improved mitochondrial function, altered mitophagy-related markers, and reduced lung injury (Sang et al., 2022), although static LC3 and p62 measurements do not establish completed mitophagic flux. In a combined LPS–peptidoglycan model, MitoQ reduced mitochondrial oxidative damage and multiorgan dysfunction (Lowes et al., 2008), but alveolar–capillary permeability and cell-specific responses were not directly assessed.

### 4.3 Human feasibility and pharmacodynamic evidence

Human evidence for MitoQ in sepsis remains preliminary. In a double-blind, randomised, placebo-controlled trial of 42 patients with septic shock, oral MitoQ at 20 mg twice daily for 5 days increased antioxidant enzyme activities and reduced malondialdehyde concentrations, but did not improve clinical or haemodynamic outcomes (Moradbaki et al., 2026). The study therefore supports oral feasibility and systemic redox pharmacodynamic activity rather than clinical efficacy. Participants were not selected for SALI/SA-ARDS or a mitochondrial-injury phenotype, and pulmonary target engagement was not assessed (Table 1). Human studies outside sepsis have shown heterogeneous biochemical, vascular, and clinical responses, indicating that systemic pharmacodynamic effects do not necessarily translate into organ-level benefit (Gane et al., 2010; Halliday et al., 2025; Y. Masoumi-Ardakani et al., 2022; Yaser Masoumi-Ardakani et al., 2022; Rossman et al., 2018; Snow et al., 2010). Human studies outside sepsis provide preliminary tolerability data, including no evident renal functional or tubular-injury signal after a single high oral dose (Linder et al., 2023) and no adverse change in kidney function or urinary kidney-injury biomarkers after 8 weeks of MitoQ supplementation (Stute et al., 2026). However, these findings cannot be directly extrapolated to critically ill patients with sepsis (Supplementary Table S1).

**TABLE 1:** MitoQ evidence relevant to acute and sepsis-associated lung injury, stratified by evidence level.

Evidence level	Study and model	MitoQ dose and timing	Main relevant outcomes	Key limitation
Direct pulmonary evidence — endothelial barrier	(Cen et al., 2021); LPS-induced lung injury in mice and LPS-treated pulmonary microvascular endothelial cells	Mice: 5 mg/kg i.p., 30 min before LPS; cells: 1 μM before LPS	Reduced mtROS, mitochondrial depolarisation, apoptosis, endothelial hyperpermeability, and vascular leakage; preserved ATP; protection involved Nrf2–MafF/ARE signalling	Predominantly preventive dosing; mtDNA-dependent signalling and pyroptotic causality were not assessed
Direct pulmonary evidence — epithelial barrier	(Hou et al., 2021); LPS-induced ALI in mice and LPS-treated A549 cells	Mice: 5 mg/kg i.p., 1 h after LPS; cells: 500 nM after LPS	Reduced mtROS, mitochondrial hyperfission, depolarisation, cytochrome c release, and caspase-3 activation; restored ZO-1 and occludin; reduced pulmonary oedema and epithelial injury	A549 cells incompletely model primary alveolar epithelium; mitochondrial apoptosis rather than pyroptosis was assessed
Direct SALI evidence — post-CLP protection	(Li et al., 2019); CLP-induced septic ALI in rats	2 mg/kg i.p. at 0 and 6 h after CLP	Improved 24-h survival; reduced pulmonary oedema, inflammatory-cell infiltration, MPO, HMGB1, IL-6, and apoptosis; LY294002 attenuated protection, supporting PI3K–Akt–GSK-3β–mTOR involvement	Short 24-h follow-up; pulmonary/mitochondrial MitoQ exposure and mtDNA-related signalling were not assessed
Direct SALI evidence — mitochondrial metabolic protection	(Sun et al., 2025); CLP-induced polymicrobial sepsis and LPS-treated macrophages	Mice: oral MitoQ 5–20 mg/kg/day for 2 weeks before CLP; macrophages: 10 μM before LPS	Improved oxygenation, respiratory-chain activity, ATP production, mitochondrial ultrastructure, and mtDNA copy number; reduced BALF protein leakage and lung injury	Prolonged pretreatment limits therapeutic relevance; mtDNA copy number does not establish abnormal mtDNA release or innate immune sensing
Other pulmonary MitoQ evidence — sterile epithelial injury	(Zhan et al., 2022); bleomycin-induced lung injury in mice and injured BEAS-2B cells	Mice: 5 mg/kg before bleomycin; cells: 25 nM	Reduced mtROS and lipid peroxidation; preserved mitochondrial membrane potential; attenuated epithelial ferroptosis and lung injury	Sterile preventive model; relevance to infection-associated SALI is indirect
Human feasibility and pharmacodynamic evidence	(Moradbaki et al., 2026); double-blind, randomised, placebo-controlled trial in 42 patients with septic shock	Oral MitoQ 20 mg twice daily for 5 days	Increased antioxidant enzyme activity and reduced malondialdehyde; no significant clinical or haemodynamic benefit	Does not establish efficacy in SALI/SA-ARDS, pulmonary exposure, mitochondrial target engagement, or pulmonary mechanistic activity

## 5 Mechanistic bridging evidence linking mitochondrial injury to alveolar–capillary barrier failure

Mechanistic studies in SALI link mitochondrial injury to abnormal mtDNA exposure, innate immune activation, regulated cell death, and alveolar–capillary barrier failure. Unless MitoQ was directly evaluated, these studies are treated as mechanistic bridging evidence rather than evidence of a MitoQ-specific mechanism (Supplementary Table S2).

### 5.1 Mitochondrial oxidative injury, mtDNA exposure, and defective quality control

Mitochondrial dysfunction in SALI is characterised by impaired respiratory-chain activity, membrane depolarisation, ATP depletion, mtROS accumulation, and lipid peroxidation, which compromise cellular bioenergetics, repair, and alveolar–capillary barrier integrity (Nagar et al., 2018; Nedel et al., 2023). The relative contribution of oxidative stress, altered mitochondrial dynamics, impaired biogenesis, and defective quality control may vary across disease stages and cell types. Mitophagy limits mtROS accumulation and the release of immunostimulatory mitochondrial constituents by removing damaged organelles. In pulmonary endothelial cells, disruption of SIRT1–Rab7-dependent late-stage mitophagy promotes dysfunctional mitochondrial retention, cytosolic mtDNA accumulation, and vascular permeability (Jiang et al., 2024). In alveolar type II epithelial cells, CerS6-mediated suppression of BNIP3-dependent mitochondrial clearance similarly increases oxidative injury and cytosolic mtDNA while impairing barrier function (Luo et al., 2026). Defects involving IRGM- or FUNDC1-dependent mitochondrial quality control have also been associated with epithelial mtDNA accumulation and cGAS–STING activation (Mi et al., 2025; Shi et al., 2025). When mitochondrial integrity or clearance fails, mtDNA may reach the cytosol or extracellular compartment through membrane permeabilisation, extracellular vesicle release, or lytic cell death (Harrington et al., 2017; Long et al., 2022).

The biological significance of mtDNA depends on its localisation, oxidation state, and abundance. Cytosolic mtDNA, circulating mtDNA, oxidised mtDNA, and mtDNA copy number represent biologically distinct readouts and should not be treated as interchangeable indicators of mitochondrial injury or danger signalling (Shimada et al., 2012; West et al., 2015; Zhong et al., 2018). Direct evidence that MitoQ modifies these processes in the injured lung remains limited. In a severe-burn model, MitoQ reduced oxidised, cytosolic, and circulating mtDNA and attenuated inflammasome-associated responses (Wu et al., 2020). Other extrapulmonary MitoQ studies reported reduced mtDNA damage or modulation of Nrf2/PINK1/Parkin-related mitochondrial quality-control pathways across intestinal, exercise-related, cardiac, renal, and cellular models (Hu et al., 2018; Ji et al., 2022; Williamson et al., 2020; Xiao et al., 2017; Zhao et al., 2025). These findings provide MitoQ-specific mechanistic plausibility but do not establish pulmonary causality.

Pulmonary studies using other interventions nevertheless support the relevance of mtDNA damage and exposure to lung injury. Enhanced repair of oxidised mtDNA reduced vascular permeability, multiorgan damage, and early mortality (Lee et al., 2017), whereas exogenous mtDNA induced pulmonary oedema and protein leakage that were attenuated by TLR9 inhibition (Zhang et al., 2016). Oxidised cytosolic mtDNA can also activate NLRP3 (Shimada et al., 2012). Whether MitoQ restores functional mitophagy in pulmonary cells remains unresolved, and static LC3 or p62 measurements cannot distinguish increased autophagosome formation from impaired lysosomal degradation or completed mitophagic flux (Sang et al., 2022). Thus, available pulmonary evidence supports causal links among defective mitochondrial quality control, abnormal mtDNA exposure, and barrier injury, but does not establish that MitoQ restores mitophagic flux or reduces cytosolic mtDNA in infection-associated SALI.

### 5.2 cGAS–STING, inflammasomes, and feed-forward inflammatory amplification

In SALI, mitochondrial injury may increase cytosolic mtDNA exposure and activate cGAS–STING signalling, although the initiating DNA source and principal responding cell type vary among experimental models. Nuclear, micronuclear, microbial, and mitochondrial DNA may all contribute to cytosolic DNA sensing. In LPS-induced acute lung injury, cytosolic mtDNA accumulation is accompanied by cGAS–STING, NLRP3, caspase-1, and GSDMD activation. Genetic deletion of cGAS or STING reduces pulmonary inflammation, tissue injury, and mortality, whereas restoration of NLRP3 activity partly reverses the protection associated with STING deficiency (Ning et al., 2020). These findings support cGAS–STING as an upstream regulator of NLRP3–GSDMD signalling in selected settings, although much of the cellular evidence is macrophage-centred and derived from LPS models rather than polymicrobial sepsis.

Human and animal studies further associate extracellular mtDNA, STING activation, and impaired mitochondrial clearance with sepsis-associated lung injury. Increased circulating mtDNA has been linked to STING activation and defective autophagic degradation, while exogenous mtDNA promotes pulmonary oedema, occludin loss, and inflammation that are attenuated by STING deficiency (Liu et al., 2021). However, circulating mtDNA does not establish cytosolic entry into a defined pulmonary cell population or direct activation of cGAS; the human evidence therefore remains associative. STING may also amplify mitochondrial injury rather than merely respond to it. STING activation can promote Ca^2+^ accumulation and recruitment of Drp1 and GSDMD-N to mitochondria, increasing mitochondrial fragmentation, membrane permeabilisation, mtROS production, and mtDNA release. STING deficiency or H-151 attenuates these responses and pulmonary oedema (Zou et al., 2025), supporting a feed-forward interaction between mitochondrial injury and STING signalling. Additional studies similarly implicate cGAS–STING in pulmonary inflammation, NLRP3 activation, GSDMD cleavage, and barrier injury (Chen et al., 2024; Comish et al., 2022; Elmorsy et al., 2026; Jia et al., 2025; Jiang et al., 2025; Ying et al., 2024; Zhou et al., 2025).

This network is not obligatorily linear. STING can be activated by non-mitochondrial DNA, while NLRP3 and GSDMD can be engaged independently of mtDNA–cGAS–STING signalling. Limited MitoQ-specific pulmonary evidence is consistent with an mtROS–NLRP3 route: in leptin-pretreated BEAS-2 cells, MitoQ reduced mtROS together with NLRP3, caspase-1, and IL-1β expression (Chong et al., 2021), but activation-specific inflammasome readouts, GSDMD cleavage, and functional pyroptotic cell death were not assessed. Cytosolic LPS may also activate inflammatory caspases and GSDMD independently of mtDNA or cGAS–STING. Current evidence therefore supports sequential, parallel, and self-amplifying pathways rather than a fixed mtDNA–cGAS–STING–NLRP3–GSDMD cascade. This network is one plausible mediator of MitoQ-associated protection, but alternative mechanisms—including preservation of mitochondrial bioenergetics and dynamics, reduced apoptosis, Nrf2-associated cytoprotection, and junctional stabilisation—remain possible. Direct rescue or epistasis experiments are required to define their relative contributions (Figure 3).

**FIGURE 3:**
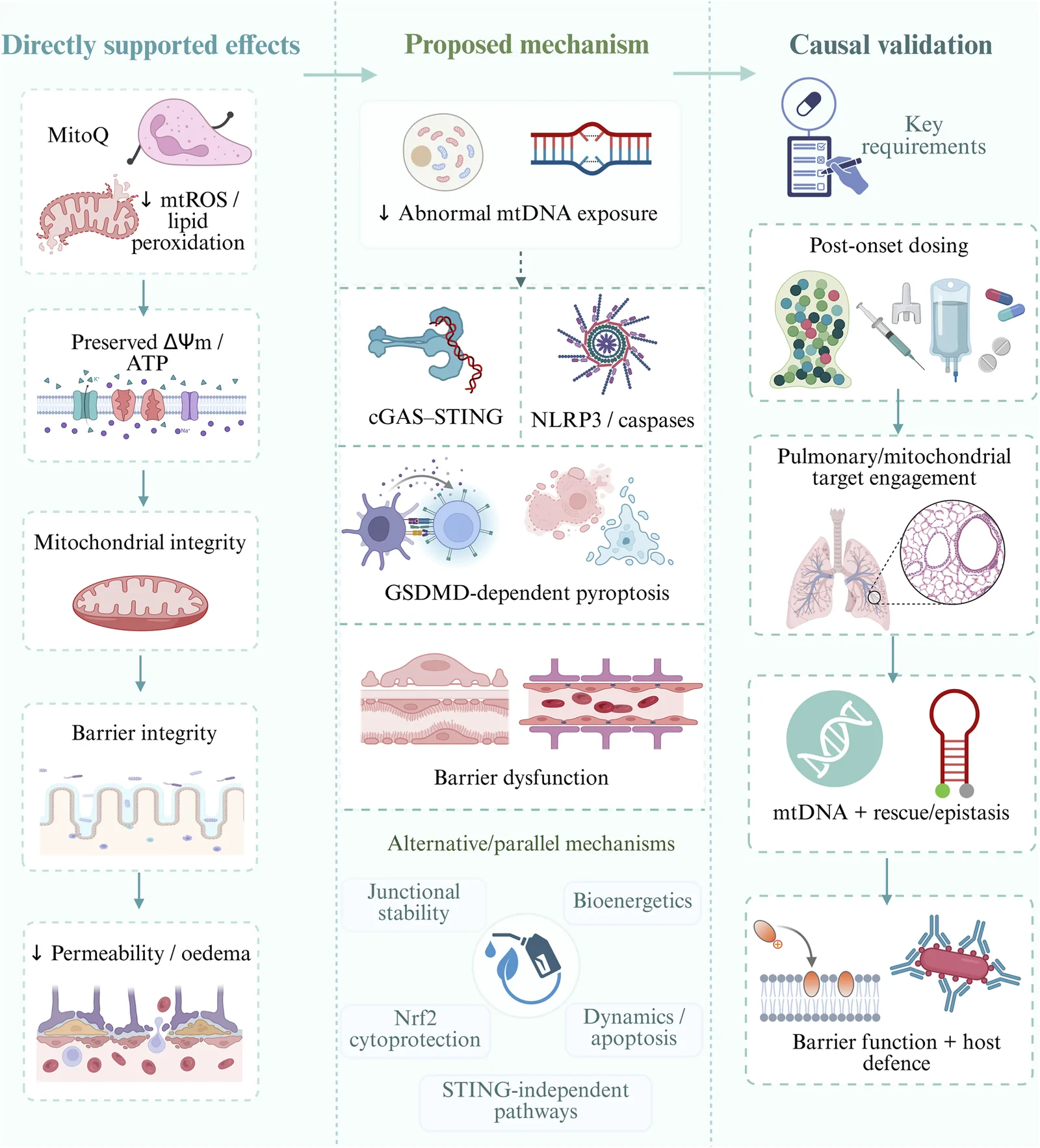
Pulmonary and sepsis-related studies directly support MitoQ-associated reduction of mitochondrial oxidative injury, preservation of mitochondrial function, and protection of epithelial, endothelial, and alveolar–capillary barrier integrity. Reduced abnormal mtDNA exposure and modulation of cGAS–STING, NLRP3/inflammatory-caspase, and GSDMD-dependent pyroptotic signalling remain proposed or indirectly supported and have not been established as mediators of MitoQ-associated pulmonary protection. Alternative or parallel mechanisms, including preservation of bioenergetics and mitochondrial dynamics, reduced apoptosis, Nrf2-associated cytoprotection, and junctional stabilisation, remain plausible. Solid arrows indicate directly supported MitoQ-associated effects; dashed arrows indicate proposed relationships.

### 5.3 Pyroptotic injury to endothelial and epithelial barriers

Pyroptosis is mediated by inflammatory caspase activation and GSDMD cleavage, generating pore-forming GSDMD-N that promotes membrane permeabilisation and lytic inflammatory cell death (Aglietti and Dueber, 2017; Booty and Bryant, 2022; Burdette et al., 2021; Kayagaki et al., 2015; Shi et al., 2015). Endothelial-specific deletion of caspase-11 reduces vascular leakage, pulmonary oedema, neutrophil recruitment, and cell death after LPS exposure or cecal ligation and puncture, supporting a causal role for caspase-11–GSDMD signalling in endothelial barrier failure (Cheng et al., 2017). Mitochondrial dysfunction may increase susceptibility to this response: SIRT3 deficiency impairs Parkin-dependent mitophagy, increases mtROS and cytosolic mtDNA, and enhances NLRP3–GSDMD signalling (Yan et al., 2025), whereas activation of the FASN–ETS1–VDAC1 axis promotes mtDNA release and subsequent cGAS–STING, caspase-1, and GSDMD-N activation (He et al., 2025). In alveolar epithelial cells, IL-17A–p38 signalling activates NLRP3, caspase-1, and GSDMD-N and increases barrier permeability (Zhou et al., 2023), while alveolar type II cell-specific Atg5 deficiency during bacterial infection impairs intracellular degradation and pathogen clearance and increases caspase-11-dependent GSDMD cleavage (Wang et al., 2025). Consistent with this non-linear framework, pyroptosis can also arise through mtDNA–STING-independent inflammatory routes (Wang Q. et al., 2024; Xie et al., 2024), whereas alveolar–capillary barrier failure may additionally result from non-pyroptotic mechanisms, including junctional disruption, cytoskeletal remodelling, altered barrier-regulatory signalling, and defective mitochondrial quality control (Bai et al., 2023; Cai et al., 2016; Chan et al., 2019; Li et al., 2023; Zhang et al., 2025). Conversely, inflammasome- and pyroptosis-associated epithelial injury contributes to selected ALI models (Chen et al., 2025; Cheng et al., 2023; Shen et al., 2024; Zhang et al., 2022). Pyroptosis should therefore be regarded as one contributor to barrier failure rather than a universal mechanism. Demonstrating pyroptosis requires activation-specific evidence. Increased total expression of NLRP3, caspase-1, GSDMD, or IL-1β alone is insufficient. Stronger evidence requires inflammatory-caspase activation, GSDMD cleavage, preferably with evidence of membrane localisation or pore formation, membrane permeabilisation, and lytic cell death. Cleavage-specific GSDMD readouts may provide additional evidence of pyroptotic activity in sepsis (Li et al., 2026). Restoration of junctional proteins or reductions in pulmonary oedema or lactate dehydrogenase release likewise do not independently demonstrate inhibition of GSDMD-mediated pyroptosis. Existing pulmonary MitoQ studies do not meet these criteria; any pyroptosis-modulating effect of MitoQ therefore remains indirect and may reflect upstream preservation of mitochondrial integrity rather than direct suppression of pyroptotic machinery.

### 5.4 Cellular crosstalk within the alveolar–capillary unit

Alveolar–capillary barrier failure involves coordinated injury across epithelial, endothelial, and immune-cell populations. These cells can interact through mtDNA-containing material, cytokines, extracellular vesicles, and other paracrine signals, propagating mitochondrial dysfunction, inflammation, and barrier injury across the alveolar–capillary unit (Bärnthaler et al., 2017; Peng et al., 2026; Wang et al., 2025). The relative contribution of individual cell populations likely varies with the infectious stimulus, disease stage, and tissue compartment. MitoQ may attenuate this cross-cellular amplification by preserving mitochondrial homeostasis and reducing mitochondrial danger signalling. However, its principal pulmonary target cells remain unknown. Current studies do not establish whether protection is mediated predominantly through endothelial cells, alveolar epithelial cells, macrophages, or recruited leukocytes, nor whether mitochondrial protection in 1 cell population is sufficient to improve neighbouring-cell function. Thus, the cell-specific and paracrine mechanisms underlying MitoQ-associated pulmonary protection remain unresolved.

## 6 Evidence tiers, causal gaps, and translational readiness

### 6.1 Directly supported effects and mechanistic boundaries

Current evidence supports MitoQ primarily as an upstream mitochondrial redox intervention that preserves mitochondrial function and alveolar–capillary barrier integrity in selected pulmonary and sepsis-related models (Cen et al., 2021; Hou et al., 2021; Li et al., 2019; Sun et al., 2025). However, the dominant downstream mediators of these effects remain unresolved. The mtDNA–cGAS–STING–NLRP3–GSDMD network is biologically plausible but has not been established as a MitoQ-specific mechanism in infection-associated SALI. MitoQ should therefore not be interpreted as a direct inhibitor of these downstream inflammatory or pyroptotic pathways.

### 6.2 Minimum experimental requirements for causal validation

Causal validation should prioritise post-onset MitoQ treatment in infection-associated SALI models with confirmed pulmonary and mitochondrial target engagement. Candidate mechanisms should be tested using compartment-specific mtDNA measurements together with pathway-directed rescue, loss-of-function, or epistasis approaches. Molecular effects should be linked to functional alveolar–capillary barrier outcomes and assessed alongside pathogen burden and clearance to determine whether mitochondrial redox modulation preserves host defence (West et al., 2011).

### 6.3 Mechanism-informed patient selection and biomarkers

SALI and sepsis-associated ARDS are biologically heterogeneous, with inflammatory and metabolic phenotypes associated with differences in prognosis and treatment response (Alipanah-Lechner et al., 2026; Calfee et al., 2014; Famous et al., 2017). No validated biomarker currently predicts responsiveness to MitoQ. Early clinical studies should therefore use biomarker-informed patient selection combining indices of mitochondrial injury with markers of epithelial and endothelial damage. Circulating mtDNA has been associated with organ dysfunction and adverse outcomes in sepsis, acute lung injury, and ARDS (Busani et al., 2020; Bushra et al., 2026; Choi et al., 2022; Faust et al., 2020; Huang et al., 2020; Wang et al., 2020; Yan et al., 2018). However, cell-free mtDNA and whole-blood mtDNA copy number represent biologically distinct measures, and methodological differences further limit cross-study comparability (Hernández-Beeftink et al., 2021; Liao et al., 2025; McClintock et al., 2022). Serial, compartment-appropriate biomarkers should therefore be related to MitoQ exposure, mitochondrial target engagement, and pulmonary injury.

### 6.4 Early-phase clinical development, exposure, outcomes, and safety

Early-phase studies should prioritise pharmacokinetics, pulmonary exposure, mitochondrial target engagement, pharmacodynamic activity, and safety. The MARVEL trial provides a precedent for biomarker-informed evaluation of oral MitoQ, although its design cannot be directly extrapolated to SALI (Gwyer Findlay et al., 2021). Dose selection should therefore be guided by disease-specific pharmacokinetic and pulmonary target-engagement data rather than regimens derived from healthy volunteers or chronic disease populations. Clinical assessment should integrate molecular biomarkers with functional pulmonary outcomes. Soluble receptor for advanced glycation end products (sRAGE) may reflect alveolar epithelial injury (Jabaudon et al., 2018), whereas angiopoietin-2 (Ang-2) is associated with endothelial injury and vascular permeability (Rosenberger et al., 2023). These biomarkers should be interpreted alongside pulmonary oedema or extravascular lung water, PaO_2_/FiO_2_, and ventilator-free days. Safety assessment should include organ toxicity, pathogen clearance, and secondary infection (West et al., 2011). Progression to efficacy testing should depend on demonstrated pulmonary target engagement, relevant pharmacodynamic activity, and acceptable safety (Figure 4; Table 2).

**FIGURE 4:**
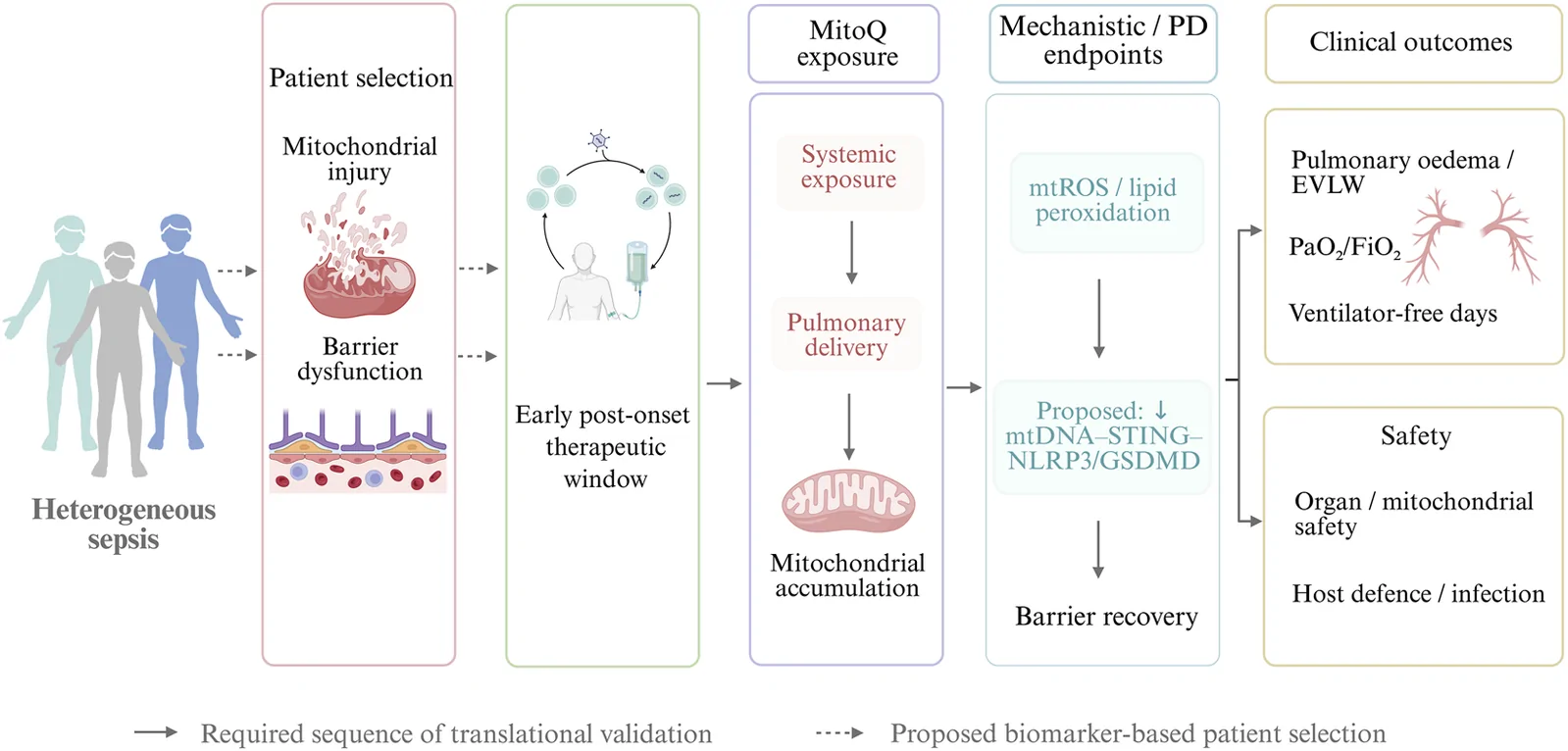
Proposed framework for the clinical development of MitoQ in SALI. Successful translation requires alignment of mechanism-informed patient selection, an early therapeutic window, adequate systemic, pulmonary, and mitochondrial exposure, mechanistic and pharmacodynamic assessment, clinically relevant outcomes, and safety evaluation. Reduced mtDNA danger-signal burden and attenuation of STING–NLRP3–GSDMD-related inflammatory signalling represent proposed mechanistic endpoints requiring MitoQ-specific validation. Solid arrows indicate the required sequence of translational validation, whereas dotted arrows indicate proposed biomarker-based patient selection.

**TABLE 2:** Translational gaps and minimum evidence required for further development of MitoQ in SALI.

Domain	Current evidence	Major uncertainty	Minimum next step
Biological rationale	Mitochondrial oxidative injury, bioenergetic failure, and mtDNA danger signalling contribute to experimental SALI; MitoQ preserves mitochondrial and barrier function in selected models	A complete MitoQ-specific mechanism linking redox modulation to reduced inflammation and barrier recovery has not been demonstrated	Test post-onset MitoQ in infection-associated SALI with temporal measurements and pathway-specific interventions
Disease-model relevance	Protection has been reported in LPS epithelial and endothelial injury and selected CLP models	LPS does not reproduce polymicrobial sepsis; CLP studies vary in species, severity, dosing, and follow-up	Use polymicrobial or bacterial infection models with standardised supportive care and longer follow-up
Treatment timing	Benefit has been reported after LPS or CLP, but several studies used prophylactic or prolonged pretreatment	Preventive dosing does not establish efficacy after clinical onset	Test post-onset dosing at clinically plausible time points and define the latest reversible window
Drug exposure and distribution	Oral dosing is feasible and systemic redox pharmacodynamic effects have been observed	Plasma, pulmonary, cellular, and intramitochondrial exposure in sepsis are unknown	Define plasma–lung–cell–mitochondrial pharmacokinetics and pharmacodynamics across disease severities
Mitochondrial target engagement	Studies report reduced mtROS and lipid peroxidation and preserved membrane potential, ATP, respiration, and ultrastructure	Effects are rarely related to tissue exposure; high concentrations may produce carrier-related or bioenergetic effects	Relate exposure to mitochondrial function and toxicity and include triphenylphosphonium carrier controls
mtDNA danger signalling	Extrapulmonary MitoQ studies suggest reduced mtDNA damage or release; other pulmonary interventions link mtDNA exposure to inflammation and lung injury	Circulating, cytosolic, oxidised, and copy-number measures are often conflated; direct effects on sensor-accessible pulmonary mtDNA are unknown	Quantify oxidised cytosolic, extracellular cell-free, and total or copy-number mtDNA separately in defined pulmonary cells
Pathway engagement and causal mechanism	cGAS–STING, inflammasomes, and GSDMD contribute to selected lung-injury models	MitoQ studies rarely assess activation-specific pathway markers or functional pyroptosis	Combine activation-specific assays with rescue or epistasis targeting mtDNA, cGAS–STING, NLRP3, inflammatory caspases, or GSDMD
Patient selection and biomarkers	mtDNA, redox, epithelial injury, and endothelial activation markers are measurable and prognostic in sepsis or ARDS	No validated biomarker predicts MitoQ response; systemic markers may not reflect pulmonary target engagement	Evaluate serial multimodal biomarkers in mechanism-enriched cohorts
Barrier and organ-level outcomes	Preclinical studies report reduced oedema, protein leakage, junctional loss, and hyperpermeability	Human pulmonary efficacy is unproven and molecular effects may not produce functional recovery	Integrate functional permeability and lung-injury measures with EVLW, PaO_2_/FiO_2_, and ventilator-free days
Safety and host defence	Available human studies suggest preliminary tolerability	Excessive mtROS suppression may impair antimicrobial signalling, pathogen clearance, metabolic adaptation, or mitochondrial function	Assess pathogen burden, clearance, secondary infection, organ function, metabolic indices, and mitochondrial toxicity
Early-phase clinical readiness	Human evidence supports dosing feasibility and systemic pharmacodynamic activity	Clinically relevant dose, target population, exposure threshold, and validated pathway remain undefined	Conduct mechanism-focused phase I/II studies linking exposure, pulmonary target engagement, pathway modulation, barrier responses, and safety

## 7 Conclusion

MitoQ has demonstrated mitochondrial and alveolar–capillary barrier protection in selected experimental models of acute and sepsis-associated lung injury, supporting its biological relevance as an upstream mitochondrial redox modulator. However, clinical efficacy in SALI remains unproven, and the mtDNA–cGAS–STING–NLRP3–GSDMD network has not been established as the principal mediator of MitoQ-associated pulmonary protection. The next priority is rigorous post-onset evaluation in infection-associated SALI with confirmed pulmonary and mitochondrial target engagement and pathway-specific causal validation.

## Glossary

ARE: Antioxidant response element
ATP: Adenosine triphosphate
BALF: Bronchoalveolar lavage fluid
BNIP3: BCL2-interacting protein 3
cGAS: Cyclic GMP–AMP synthase
CLP: Cecal ligation and puncture
Drp1: Dynamin-related protein 1
EVLW: Extravascular lung water
GSDMD: Gasdermin D
GSDMD-N: Gasdermin D N-terminal fragment
GSK-3β: Glycogen synthase kinase 3 beta
HMGB1: High-mobility group box 1
IL: Interleukin
IRF3: Interferon regulatory factor 3
LPS: Lipopolysaccharide
MafF: Musculoaponeurotic fibrosarcoma oncogene homolog F
MitoQ: Mitoquinone
mtDNA: Mitochondrial DNA
mtROS: Mitochondrial reactive oxygen species
mTOR: Mechanistic target of rapamycin
NLRP3: NOD-, LRR- and pyrin domain-containing protein 3
Nrf2: Nuclear factor erythroid 2-related factor 2
**PaO**_**2**_ **/FiO**_**2**_: Ratio of arterial oxygen partial pressure to the fraction of inspired oxygen
PI3K: Phosphoinositide 3-kinase
PINK1: PTEN-induced kinase 1
PMVECs: Pulmonary microvascular endothelial cells
SA-ARDS: Sepsis-associated acute respiratory distress syndrome
SALI: Sepsis-associated acute lung injury
SIRT1: Sirtuin 1
STING: Stimulator of interferon genes
TBK1: TANK-binding kinase 1
TEER: Transepithelial electrical resistance
TFAM: Mitochondrial transcription factor A
TLR9: Toll-like receptor 9
**TPP** ^**+**^: Triphenylphosphonium cation
ZO-1: Zonula occludens 1
