# Supplementary material for: MitoQ in sepsis-associated acute lung injury: redox pharmacology, mechanistic evidence, and translational perspectives
Source: Front Pharmacol. 2026 Sep 30;17:1975929. doi: 10.3389/fphar.2026.1975929 (PMC13661397; doi:10.3389/fphar.2026.1975929)
Supplement: Supplementary file 1 [file Supplementaryfile1.docx]

**Supplementary Table S1.** Additional human and extrapulmonary MitoQ evidence relevant to safety, translational interpretation, and mechanistic plausibility

| **Evidence context** | **Study/model** | **MitoQ regimen** | **Main finding** | **Relevance to SALI** |
| --- | --- | --- | --- | --- |
| Human safety | (Linder et al., 2023); healthy adults | Single oral dose, 100–160 mg | No significant adverse renal functional or tubular-injury signal | Supports preliminary short-term human tolerability but does not establish safety in critically ill sepsis |
| Human translational context | (Halliday et al., 2025); dilated cardiomyopathy | 40 mg/day for 3–12 months | No improvement in myocardial energetics or clinical functional outcomes | Illustrates that prolonged systemic exposure does not necessarily translate into organ-level efficacy |
| Extrapulmonary MitoQ mechanistic evidence — mtDNA/NLRP3 | (Wu et al., 2020); severe-burn model and macrophages | Rats: 4 mg/kg; cells: 1 μM | Reduced oxidised, cytosolic and circulating mtDNA and attenuated NLRP3-associated responses | Supports the possibility that MitoQ can modify abnormal mtDNA exposure, but pulmonary relevance is unproven |
| Extrapulmonary MitoQ mechanistic evidence — mtDNA/barrier | (Hu et al., 2018); intestinal ischaemia–reperfusion | Animals: 4 mg/kg; cells: 0.1–1 μM | Reduced mtDNA damage and preserved TFAM, tight-junction proteins and intestinal permeability | Provides MitoQ-specific plausibility linking mitochondrial genome preservation with epithelial barrier protection |
| Extrapulmonary MitoQ mechanistic evidence — quality control | (Xiao et al., 2017); diabetic renal/metabolic models | Mice: 5 mg/kg twice weekly for 12 weeks | Restored Nrf2/PINK1–Parkin signalling, reduced damaged mitochondria and improved ATP production | Supports effects of MitoQ on mitochondrial quality control outside the lung |
| Human safety — extended exposure | (Stute et al., 2026); middle-aged and older adults | 20 mg/day for 8 weeks; randomized placebo-controlled crossover study | No meaningful change in glomerular filtration measures or urinary kidney-injury biomarkers | Extends preliminary human renal tolerability beyond single-dose exposure, but does not establish safety in critically ill sepsis |

**Supplementary Table S2.** Mechanistic bridging evidence linking mitochondrial danger signalling, innate immune activation, pyroptosis, and alveolar–capillary barrier dysfunction in SALI

| **Mechanistic link and study** | **Model/cell type** | **Causal test(s)** | **Key findings** | **Mechanistic relevance and key limitations** |
| --- | --- | --- | --- | --- |
| Mitophagy failure–mtDNA–STING/NLRP3 (Jiang et al., 2024) | CLP-SALI mice; LPS-treated pulmonary endothelial cells | SIRT1/Rab7 modulation; mtDNA depletion; cGAS/STING/NLRP3/GSDMD inhibition | Defective mitophagy increased mtROS, cytosolic mtDNA, STING/NLRP3 activation, vascular leakage, and oedema | Supports an endothelial mitophagy–mtDNA–barrier axis; several interventions were preventive/pharmacological |
| CerS6/BNIP3–mtDNA–STING/NLRP3 (Luo et al., 2026) | LPS/CLP lung injury; alveolar type II cells | CerS6 deletion; BNIP3 knockdown | Impaired mitophagy increased cytosolic mtDNA and STING/NLRP3 activity; CerS6 deletion preserved barrier integrity | Supports an epithelial mitophagy–mtDNA–barrier axis; GSDMD-mediated pyroptosis remains uncertain |
| mtDNA–cGAS–STING–NLRP3/GSDMD (Ning et al., 2020) | LPS-ALI mice; primary macrophages | cGAS/STING deficiency; mtDNA manipulation; NLRP3 overexpression; STING agonism | Cytosolic mtDNA activated cGAS–STING–NLRP3/GSDMD; pathway disruption reduced lung injury and mortality | Strong causal support for the axis, but mainly in macrophage/LPS models |
| Circulating mtDNA–STING–autophagic dysfunction (Liu et al., 2021) | Patients with SALI; LPS/CLP and mtDNA mouse models; macrophages | STING modulation; exogenous mtDNA; autophagy/TBK1–NF-κB interference | mtDNA–STING impaired autophagic degradation and aggravated mitochondrial and barrier injury | Links extracellular mtDNA to impaired mitochondrial clearance; human evidence was associative |
| Mitochondrial danger signals–STING (Elmorsy et al., 2026) | LPS-ALI mice | Elamipretide; H-151; combined treatment | Mitochondrial protection reduced ROS, lipid peroxidation, and cytosolic mtDNA; STING inhibition reduced inflammation | Supports upstream mitochondrial control of STING; GSDMD-mediated pyroptosis was not assessed |
| STING–Ca²⁺–Drp1/GSDMD-N–mtDNA feedback (Zou et al., 2025) | LPS-ALI; STING-knockout mice; macrophage models | STING inhibition; disulfiram; Mdivi-1; Ca²⁺ blockade | STING promoted Drp1/GSDMD-N recruitment, mitochondrial injury, mtROS, and mtDNA release | Supports a feed-forward STING–GSDMD–mitochondrial injury loop; mainly macrophage-based |
| STING palmitoylation–epithelial pyroptosis (Jia et al., 2025) | CLP-SALI; epithelial cells; septic human lung tissue | ZDHHC21/ETS2 modulation; AUF1 overexpression; inflammasome inhibition | STING palmitoylation promoted caspase-1 activation, GSDMD-N formation, and epithelial pyroptosis | Links STING to epithelial pyroptosis; mtROS/mtDNA were not established as initiating signals |
| Caspase-4/5/11–GSDMD endothelial pyroptosis (Cheng et al., 2017) | Endothelial cells; LPS endotoxaemia; CLP | Caspase-4/5 knockdown; caspase-11 deletion | Cytosolic LPS induced GSDMD-dependent endothelial lysis, leakage, and oedema | Establishes an mtDNA–STING-independent route to endothelial barrier failure |
| IL-17A/p38–NLRP3/GSDMD epithelial injury (Zhou et al., 2023) | Sepsis samples; Th17–A549 coculture; CLP mice | Th17, p38, or NLRP3 inhibition | IL-17A promoted NLRP3/GSDMD-dependent pyroptosis and epithelial hyperpermeability | Demonstrates an mtDNA-independent pyroptotic pathway; A549 cells limit extrapolation |
| Exosomal mtDNA–TLR9/PKCδ endothelial dysfunction (Peng et al., 2026) | Sepsis-derived exosomes; mtDNA-treated endothelial cells | TLR9 or PKCδ knockdown | Exosomal mtDNA impaired mitochondrial function, TEER, and junctional integrity through TLR9–PKCδ | Demonstrates a cGAS–STING-independent mtDNA-sensing pathway; pyroptosis was not established |

**Note:** These studies provide mechanistic bridging evidence rather than direct evidence of MitoQ action in SALI. MitoQ was not directly tested in the studies summarised here; therefore, the findings support biological plausibility and pathway-specific causality but do not establish a MitoQ-specific mechanism.

Cheng, K. T., Xiong, S., Ye, Z., Hong, Z., & Malik, A. B. (2017). Caspase-11–mediated endothelial pyroptosis underlies endotoxemia-induced lung injury. *Journal of Clinical Investigation*, *127*(11), 4124.

Elmorsy, E. A., Amer, M. M., Hamad, R. S., Abdel-Hamed, M. R., Farrag, A. A., Alorini, M., Khodeir, M. M., Ali, M. A. M., Alhowail, A. H., Mohamed, E. A., Alsalloom, A. A., Alsaykhan, H., Alsoqih, N. S., Ata, H. S., Sameh, A., Chaudhary, A. A., & Saber, S. (2026). Breaking a mitochondrial danger–STING feed-forward amplifier preserves alveolar–capillary architecture and dampens interferon–chemokine signaling in acute lung injury. *Tissue and Cell*, *101*, 103514. <https://doi.org/https://doi.org/10.1016/j.tice.2026.103514>

Halliday, B. P., Owen, R., Ragavan, A., Smith, K. L., Statton, B. K., Berry, A., Kasiakogias, A., Tsoumani, Z., Shanmugnathan, M., Dungu, J. N., de Marvao, A., Tayal, U., Ware, J. S., O’Regan, D. P., Pennell, D. J., Cleland, J. G. F., Prasad, S. K., Gregson, J., Murphy, M. P.,…Valkovič, L. (2025). A double-blind, randomised placebo-controlled trial examining the effect of MitoQ on myocardial energetics in patients with dilated cardiomyopathy. *European heart journal. Cardiovascular Imaging*.

Hu, Q., Ren, J., Li, G., Wu, J., Wu, X., Wang, G., Gu, G., Ren, H., Hong, Z., & Li, J. (2018). The mitochondrially targeted antioxidant MitoQ protects the intestinal barrier by ameliorating mitochondrial DNA damage via the Nrf2/ARE signaling pathway. *Cell Death Dis*, *9*(3), 403. <https://doi.org/10.1038/s41419-018-0436-x>

Jia, M., Peng, F., Xu, P., Fan, Y., Liu, J., Luo, B., Wu, W., Wang, Y., Zhang, Z., & Wang, Y. (2025). Sepsis-induced acute lung injury: AUF1 regulates pyroptosis via ETS2/ZDHHC21-mediated STING palmitoylation : A therapeutic target for lung injury. *Cell Mol Life Sci*, *83*(1), 48. <https://doi.org/10.1007/s00018-025-06038-4>

Jiang, T., Liu, E., Li, Z., Yan, C., Zhang, X., Guan, J., Zhan, Y., Zhao, B., & Ding, W. (2024). SIRT1-Rab7 axis attenuates NLRP3 and STING activation through late endosomal-dependent mitophagy during sepsis-induced acute lung injury. *Int J Surg*, *110*(5), 2649–2668. <https://doi.org/10.1097/js9.0000000000001215>

Linder, B. A., Stute, N. L., Hutchison, Z. J., Barnett, A. M., Tharpe, M. A., Kavazis, A. N., Kirkman, D. L., Gutierrez, O., & Robinson, A. T. (2023). Acute High Dose MitoQ Does not Increase Urinary Kidney Injury Markers in Healthy Adults. *American journal of physiology. Renal physiology*.

Liu, Q., Wu, J., Zhang, X., Li, X., Wu, X., Zhao, Y., & Ren, J. (2021). Circulating mitochondrial DNA-triggered autophagy dysfunction via STING underlies sepsis-related acute lung injury. *Cell Death & Disease*, *12*(7), 673. <https://doi.org/10.1038/s41419-021-03961-9>

Luo, G., Li, D., Wang, Z., Li, G., Zuo, Y., Jiang, W., Liu, Y., Li, B., Xie, X., Li, N., & Liu, B. (2026). CerS6 orchestrates mitochondrial-immune crosstalk via BNIP3-dependent mitophagy suppression and mtDNA-STING/NLRP3 activation in acute lung injury. *Cell Mol Life Sci*. <https://doi.org/10.1007/s00018-026-06227-9>

Ning, L., Wei, W., Wenyang, J., Rui, X., & Qing, G. (2020). Cytosolic DNA-STING-NLRP3 axis is involved in murine acute lung injury induced by lipopolysaccharide. *Clin Transl Med*, *10*(7), e228. <https://doi.org/10.1002/ctm2.228>

Peng, Z., Cao, X., Gao, H., Li, C., Zhang, J., & Liu, Y. (2026). Exosome-derived mtDNA disrupts endothelial barrier integrity and accelerates sepsis progression by inducing mitochondrial dysfunction through the PKCδ gene. *Biochim Biophys Acta Gen Subj*, *1870*(1), 130871. <https://doi.org/10.1016/j.bbagen.2025.130871>

Stute, N. L., Muma, J. P., Hutchison, Z. J., Culver, M. N., Linder, B. A., Sanchez, S. O., Kirkman, D. L., Gutierrez, O. M., Kavazis, A. N., & Robinson, A. T. (2026). Eight weeks of MitoQ supplementation does not alter kidney function or urinary kidney injury biomarkers in middle-aged and older adults. *Am J Physiol Renal Physiol*, *331*(2), F272–f285. <https://doi.org/10.1152/ajprenal.00174.2026>

Wu, Y., Hao, C., Liu, X., Han, G., Yin, J., Zou, Z., Zhou, J., & Xu, C. (2020). MitoQ protects against liver injury induced by severe burn plus delayed resuscitation by suppressing the mtDNA-NLRP3 axis. *International Immunopharmacology*, *80*, 106189. <https://doi.org/https://doi.org/10.1016/j.intimp.2020.106189>

Xiao, L., Xu, X., Zhang, F., Wang, M., Xu, Y., Tang, D., Wang, J., Qin, Y., Liu, Y., Tang, C., He, L., Greka, A., Zhou, Z., Liu, F., Dong, Z., & Sun, L. (2017). The mitochondria-targeted antioxidant MitoQ ameliorated tubular injury mediated by mitophagy in diabetic kidney disease via Nrf2/PINK1. *Redox Biol*, *11*, 297–311. <https://doi.org/10.1016/j.redox.2016.12.022>

Zhou, K. L., He, Y. R., Liu, Y. J., Liu, Y. M., Xuan, L. Z., Gu, Z. Y., He, H. Y., & Ju, M. J. (2023). IL-17A/p38 Signaling Pathway Induces Alveolar Epithelial Cell Pyroptosis and Hyperpermeability in Sepsis-Induced Acute Lung Injury by Activating NLRP3 Inflammasome. *Advanced biology*, *7*(12), e2300220. <https://doi.org/10.1002/adbi.202300220>

Zou, S., Zuo, Y., Chen, Y., Zhang, T., Fu, T., Li, G., Xiong, R., Liu, B., Hu, Y., Hu, Z., Miao, C., Wu, X., Li, N., & Geng, Q. (2025). Inhibition of STING-induced mitochondrial Drp1/N-GSDMD-mediated MtDNA release alleviates Sepsis-induced lung injury. *Cell Mol Life Sci*, *82*(1), 305. <https://doi.org/10.1007/s00018-025-05774-x>
